# Designing the Surface Chemistry of Inorganic Nanocrystals for Cancer Imaging and Therapy

**DOI:** 10.3390/cancers14102456

**Published:** 2022-05-16

**Authors:** Fanny Delille, Yuzhou Pu, Nicolas Lequeux, Thomas Pons

**Affiliations:** 1 Laboratoire de Physique et d’Etude des Matériaux, Ecole Supérieure de Physique et Chimie Industrielle, Université PSL (Paris Sciences & Lettres), Centre National de Recherche Scientifique, 75005 Paris, France; fanny.delille@espci.fr (F.D.); yuzhou.pu@espci.fr (Y.P.); nicolas.lequeux@espci.fr (N.L.); 2 Laboratoire de Physique et d’Etude des Matériaux, Centre National de Recherche Scientifique, Sorbonne Université, 75005 Paris, France

**Keywords:** nanoparticle, gold, iron oxide, quantum dot, surface chemistry, cancer imaging, therapy

## Abstract

**Simple Summary:**

Inorganic nanocrystals such as gold, iron oxide and semiconductor nanocrystals have intrinsic optical or magnetic properties that make them very promising for cancer detection, imaging and therapy. The surface ligands of these nanoparticles play a critical role in their application and control the nanoparticle interaction with biomolecules and cells. The complex nano-bio interface is, for this reason, gaining increasing attention, and many studies are devoted to either propose new surface chemistries or characterize their impact on nanoparticle imaging and/or therapeutic efficiency. This review presents a general perspective on the design of nanoparticle surface chemistry.

**Abstract:**

Inorganic nanocrystals, such as gold, iron oxide and semiconductor quantum dots, offer promising prospects for cancer diagnostics, imaging and therapy, due to their specific plasmonic, magnetic or fluorescent properties. The organic coating, or surface ligands, of these nanoparticles ensures their colloidal stability in complex biological fluids and enables their functionalization with targeting functions. It also controls the interactions of the nanoparticle with biomolecules in their environment. It therefore plays a crucial role in determining nanoparticle biodistribution and, ultimately, the imaging or therapeutic efficiency. This review summarizes the various strategies used to develop optimal surface chemistries for the in vivo preclinical and clinical application of inorganic nanocrystals. It discusses the current understanding of the influence of the nanoparticle surface chemistry on its colloidal stability, interaction with proteins, biodistribution and tumor uptake, and the requirements to develop an optimal surface chemistry.

## 1. Introduction

Nanoparticles (NPs) are defined as having sizes in the range of a few nanometers to a few hundreds of nanometers. They play a major role, and continue to be actively developed, for nanomedicine applications. Among these, organic NPs constitute the most important proportion, with polymeric and lipid NPs being used, for example, in drug delivery and vaccination. The nature and composition of these NPs can be precisely tuned using organic and macromolecular chemistry. Inorganic materials constitute other types of materials with interesting intrinsic properties. Amorphous materials, such as silica, have strong potential as drug carriers. On the other hand, inorganic nanocrystals (NCs) present a set of intrinsic photophysical and magnetic properties that can be harnessed to design novel preclinical and clinical imaging contrast agents and therapeutic modalities [1]. Two prominent examples of inorganic NCs developed for imaging and therapy are iron oxide NPs, for their superparamagnetic properties, and gold NPs, for their plasmonic properties providing strong light absorption. Even though many studies are still devoted to the optimization of their magnetic and optical properties, the synthesis schemes of these NCs are now sufficiently developed to finely adapt these properties to specific applications. For example, controlling gold NP size and shape enables tuning the wavelength of their plasmon resonance into the near infrared range, where light penetrates deeper into biological tissues than in the visible range. The size and shape of iron oxide NPs can also be tuned to maximize the contrast provided in different modes of magnetic resonance imaging (MRI). However, controlling the interactions between the NPs and their complex biological environment currently remains a challenge. In vivo, these NPs are typically injected intravenously to reach their tumor target tissue. After injection, the NPs become surrounded by a large variety of biomolecules, including lipids, peptides, and proteins, which can interact in a nonspecific and hardly predictable manner with the NP surface. These interactions control the fate of the NPs and can have dramatic adverse consequences ranging from NP agglomeration to rapid capture by immune cells. This strongly impedes their ability to reach the target tissue and deliver the expected imaging and/or therapeutic capability. There is therefore an increasing effort to understand interactions at the nano/bio interface, to design NP surface chemistries that are able to overcome these obstacles and to optimize them for specific applications. The NP surface chemistry focuses on the layer which surrounds the inorganic NP. It is usually composed of organic molecules and polymers, called ligands, which are bound to the NP surface. It ensures the colloidal stability and controls the interactions with biomolecules from the environment. As such, it plays a central role in the NP performance, both in imaging and therapeutic applications, but also on their safety for healthy cells and tissues [2,3,4]. This review focuses on the most commonly used types of inorganic nanocrystals used for cancer imaging and therapy, such as gold, iron oxide and semiconductor quantum dots, but the discussion remain valid for other types of inorganic NPs as well. First, the main physico-chemical properties of these inorganic NPs are presented. Their applications for cancer imaging and therapy are rapidly discussed to provide context, and the reader is referred to previously published reviews for more complete description [5,6,7,8,9,10,11]. Then, the different strategies used to coat these NPs for biological applications are discussed as well as the choice of ligands and how bind them to the NP surface. The interactions of biomolecules, and in particular proteins, with the NP surface are discussed. Finally, the role of the surface chemistry in determining the NP in vivo biodistribution and ultimately imaging and therapeutic efficiency is presented.

## 2. Inorganic Nanocrystals for Cancer Imaging and Therapy

### 2.1. Gold NPs

**Figure 1:**
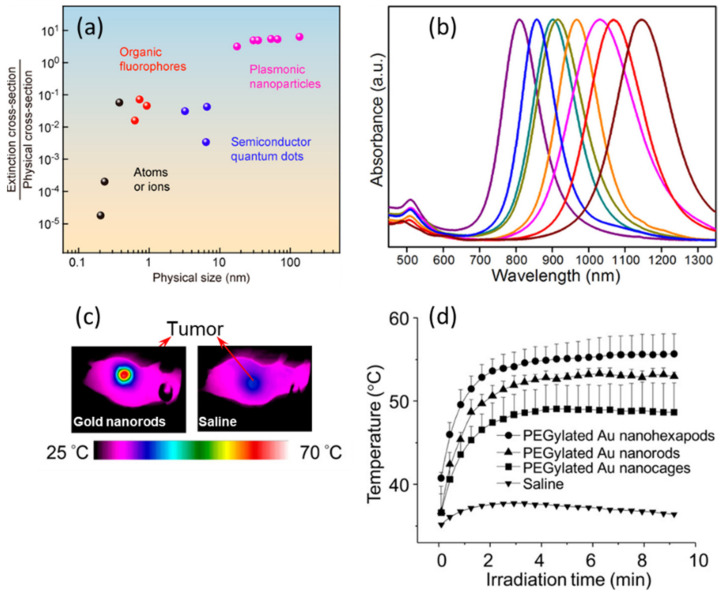
(**a**) Among various types of NPs, metallic plasmonic NPs possess the highest ratio between extinction cross-section and their physical cross-section. (**b**) The longitudinal absorption of gold nanorods can be precisely controlled during the synthesis and can extend towards near infrared region. Longitudinal absorptions of AuNR shift from 780 nm to 1200 nm as aspect ratios of AuNR increase from 3.8 to 7.5. (**c**) Thermographs of tumor-bearing mice receiving photothermal therapy. (**d**) Temperature increase within tumor region as a function of irradiation time. A diode laser (808 nm) at a power density of 1.2 W/cm² was applied for these experiments. Reproduced with permission from the American Chemical Society [18,41,42].

Noble metal NPs, in particular gold NPs, in the context of biological diagnostics and therapy, can effectively convert the energy of incident light into a collective oscillation of the electrons within the NP. This light absorption is particularly efficient at wavelengths corresponding to resonances. These resonances are called localized surface plasmonic resonances and their wavelengths depend on the NP size, shape, material composition and surrounding medium [12,13,14]. Different strategies enable the synthesis of gold NPs with different shapes (nanorods [15], nanocage [16], nanocubes [17]) extending the resonance from the visible to near infrared range (typically 520–600 nm for spheres, 600–1300 nm for nanorods, Figure 1a) where light can penetrate deeper into biological tissues. At the resonance wavelength, gold NPs are the type of material most efficient at absorbing and scattering light (Figure 1b) [18] This provides them with a distinct color and enables sensitive detection by the naked eye. This property has been used extensively for biomedical diagnostics, typically in immunogenic strip tests [19].

Gold NPs are relatively chemically inert, which makes them highly biocompatible and has driven the development of their applications as in vivo diagnostic and therapeutic agents [3,20]. The collective oscillation of surface electrons at the surface of gold NPs under irradiation can amplify the Raman scattering signals of molecules attached at the surface of gold NPs, with a signal enhancement about 100 times higher than that from the original non-conjugated organic substance [21]. Although Raman spectroscopy is a nondestructive strategy used to reveal molecular structure information and vibration with high sensitivity and rapidity, its applications are always restricted by low signal intensity, lack of appropriate probes and photobleaching of detectors [22]. Due to their unique surface-enhance Raman scattering (SERS) effect, gold NPs combined with specific immune probes or antibodies significantly increase sensitivity and contrast of detection, extending irradiation towards the near infrared region, and are stable during repetitive diagnostics, and, therefore, are important imaging agents for cancer cell identification and imaging [23,24,25]. The absorption of light by gold NPs at the plasmonic resonance increases the temperature both inside and around NPs, leading to thermoelastic expansion around them. This expansion creates a pressure transient that can be detected acoustically [26,27]. This light-in–sound out approach, called photoacoustic imaging (PAI), not only overcomes the low depth penetration caused by scattering in tissue for optical imaging, but also solves the poor spectral capability limits and low contrast of ultrasound imaging (Figure 1c). In addition, gold NPs have also been widely studied and applied as potential nanomedicines for phototherapy against cancers. The heat dissipating from NPs under high illumination results in sufficient local increase of temperature to elicit cancer cell death; the increase in the local tumor temperature increase can reach more than 10 °C upon illumination (Figure 1d). This photothermal therapy (PTT) method has been optimized with different types of gold NPs [28,29,30], surface modifications [31,32], and therapy combinations [33,34]. Apart from heat generation, high power illumination of gold NPs may also produce reactive oxygen species (ROS) such as singlet oxygen and hydroxyl radicals, which can efficiently elicit tumor cell apoptosis. This photodynamic therapy (PDT) strategy works under NIR light excitation [35,36], making it promising for in vivo applications. The efficiency of ROS production is quite low, but could be improved by the formation of hybrid NPs of gold functionalized with inorganic materials [37] or organic molecules [38]. Finally, gold atoms have a high X-ray absorption cross-section, which makes them potential X-ray computed tomography (CT) imaging probes [39,40]. However, high concentrations of gold are often required to provide sufficient contrast.

### 2.2. Magnetic NPs

**Figure 2:**
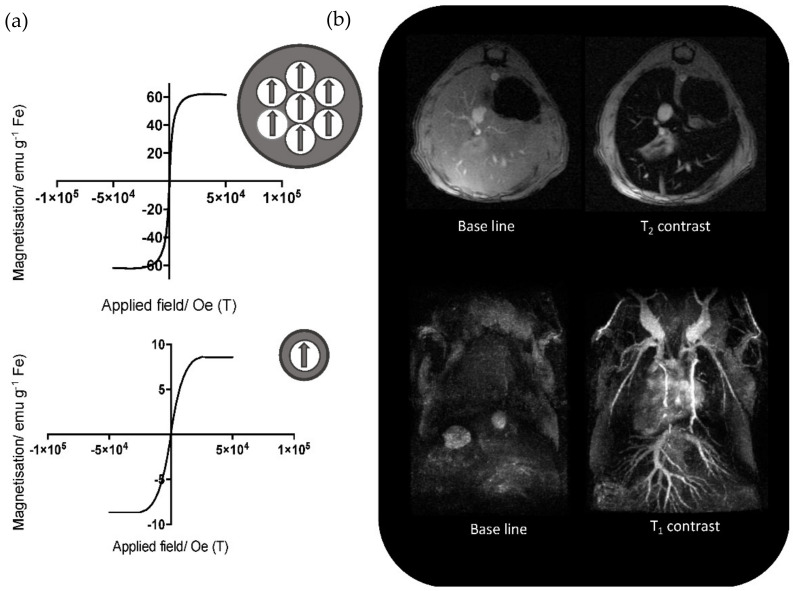
(**a**) Magnetic moment per gram of iron of larger (top) and smaller (bottom) IO-MNPs (note the change of scale). (**b**) Mouse liver T2-MRI using larger IO-MNPs (top) and T1-MRI angiography using smaller IO-MNPs. Reproduced with permission from MDPI [50].

Magnetic NPs (MNPs) are one of the most commonly used inorganic nanocrystals for biomedical applications. Among the different magnetic materials that can be used as NPs, iron oxide (IO) is the most prominent, thanks to its easy fabrication and biocompatibility. The magnetic properties of IO-MNPs originate from the interactions and relative orientations of the magnetic spins from the iron ions, and therefore strongly depends on the composition and crystalline phase of iron oxide. This exists in several magnetic phases, such as maghemite, γ-Fe_2_O_3_, containing Fe^3+^, or magnetite, Fe_3_O_4_, containing both Fe^2+^ and Fe^3+^. Other phases, such as hematite, α-Fe_2_O_3_, display much weaker magnetic properties. Alloys of IO with other metal cations such as zinc or manganese ions have also been developed to improve their magnetic properties. Different shapes (spheroid, cube, octopod) and size of IO-MNPS can be synthesized, with different magnetic properties. Larger micron-size magnetic particles display a strong permanent magnetization and are commonly used to perform magnetic separation in analytical applications, to isolate specific biomolecules or cells. Magnetic NPs (typically 5–50 nm) also have strong permanent magnetic dipoles, whose orientation, however, fluctuates rapidly in the absence of an external magnetic field. They are also valuable tools for several biomedical applications, such as magnetic resonance imaging (MRI), an imaging technique that is based on the measurement of the nuclear spin relaxation of hydrogen from water molecules (Figure 2). Through the interaction with its local magnetic field, a magnetic NP changes the relaxation time of hydrogen nuclei around it and can act as a contrast agent. Several magnetic NPs are on the market as T2 “negative” contrast agents for MRI, consisting of either single small (20–40 nm) IO-MNPs or as larger clusters (80–150 nm) containing several IO-MNPs (Feridex^®^, Endorem™, GastroMARK^®^, Lumirem^®^, Resovist^®^) [43,44]. Smaller (2–3 nm) iron oxide NPs are also being developed as T1 “positive” contrast agents [45]. Magnetic NPs also have the property of producing heat when an external alternating magnetic field is applied, called magnetic hyperthermia. In the external magnetic field, magnetic NPs elevate the temperature of their surrounding by a few degrees which can cause tumor cell death by cell apoptosis. [46,47,48,49] Magnetic NPs can be used for several applications together, which makes them a theranostic agent, i.e., a product that can be useful both in therapy and diagnosis.

### 2.3. Semiconductor Quantum Dots

**Figure 3:**
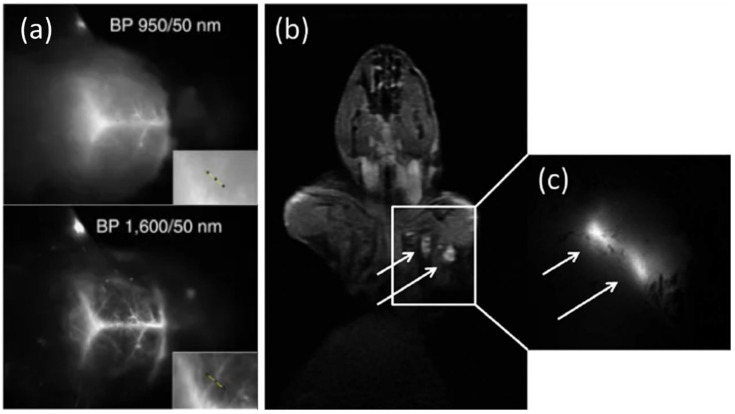
(**a**) Fluorescence imaging of the brain vasculature through intact skin and skull using SWIR emitting QDs with bandpass (BP) filters centered around 950 nm and 1600 nm, showing the improvement in spatial resolution at longer wavelengths. Reproduced with permission from Nature Publishing Group [48]. (**b**) Multimodal detection of lymph nodes by T1-weighted MRI. (**c**) NIR fluorescence using subcutaneously injected Mn^2+^-doped CuInSe_2_ QDs. Reproduced with permission from the Royal Society of Chemistry [66].

Semiconductor nanocrystals, also called Quantum Dots (QDs), display optical properties that make them valuable fluorescence emitters [51]. They have a broad absorption spectrum and high extinction coefficients, as well as emission spectra that can be tuned with respect to size and composition. In the visible range, they are usually composed of II-VI materials such as CdSe/ZnS core/shell heterostructures, or III-V compounds such as InP (roman numerals refer to columns of the periodic table, for example II corresponds to Zn and Cd). Their brightness and photostability have been leveraged in many applications [4], for example, to design high sensitivity bio-detection schemes and bright probes for single biomolecule tracking in live cells [52]. They offer bright nanoprobes in the near infrared range (NIR, typ. 700–1000 nm), where scattering and absorption of light by tissues is much reduced compared to the visible [53]. NIR-emitting QDs can be composed of potentially toxic materials such as CdTe, InAs, HgS, and PbS, or of more biocompatible CuInS_2_ or CuInSe_2_ materials, which display a much lower toxicity, making them promising for applications such as fluorescence-guided surgery [54]. They have been demonstrated in particular for tumor imaging [55], fluorescence detection of regional and sentinel lymph nodes [54,56,57,58], intratumoral imaging of blood vasculature [59] and detection of circulating tumor cells [60]. In the short-wave infrared range (SWIR, 1000–1700 nm), light penetrates even deeper into tissues, with a lower fluorescence background and much reduced scattering. This enables preservation of good spatial resolution and contrast deep into tissues (Figure 3a). Semiconductor QDs constitute one of the brightest class of SWIR emitting materials, with QDs composed of InAs, PbS, but also low toxicity Ag_2_S [53,61,62]. Quantum dots are well suited for designing multimodal imaging agents to combine the high spatial resolution, ease of use and sensitivity of fluorescence detection with the whole body and 3D imaging capability of MRI or PET imaging (Figure 3b,c) [63,64,65,66]. Finally, the strong absorbance of QDs makes them interesting materials to explore for the production of radical oxygen species upon illumination using visible or near infrared light, and for photodynamic therapy [67]. The clinical application of QDs has been limited, so far, to ex vivo diagnostics without any application to human patients, but their use in cellular and preclinical small animal studies is rapidly increasing.

### 2.4. Inorganic Nanocrystals as Therapeutic Enhancers

Beyond the use of the specific plasmonic, magnetic or fluorescent properties of the above-mentioned NPs for diagnostics, imaging or therapy, there are other interesting applications which have emerged for cancer therapy and which share the same challenges regarding functionalization and surface chemistry. For example, the use of inorganic nanocrystals as nanocarriers for drug delivery presents various interesting specificities. Inorganic nanocarriers such as mesoporous silica [68], metal-organic framework [69,70] nanocrystals such as calcium phosphate, gold, iron oxide and quantum dots, have been studied for drug delivery [71,72]. They may have better stability against biodegradation than organic nanocarriers and may enable a more favorable biodistribution for drugs. In particular, they may slow down renal excretion, prevent biodegradation by enzymes and enable a better tumor uptake. Moreover, the specific properties of inorganic nanocrystals may be harnessed to improve drug delivery in several ways. For example, magnetic NPs can be used as drug carriers. When drugs are loaded onto the magnetic NPs they can be concentrated locally in a targeted tissue using an externally applied magnetic gradient and released in a controlled way under stimuli such as heat, pH, or a magnetic field [73,74,75]. Gold NPs may also be used to trigger the release of conjugated DNA or drug molecules due to the production of heat upon illumination [76,77]. Since these nanocrystals are also contrast agents for MRI, photoacoustic and fluorescence imaging, they also provide a way to evaluate, in real time, the biodistribution of the drug after injection and the efficiency of tumor uptake. Finally, NPs can be incorporated into larger assemblies (liposomes, polymersomes, and others) and used to trigger drug release from larger carriers [34,78,79].

Another interesting property of inorganic NPs arises from the radio-sensitization effect. Radiotherapy relies on direct DNA damage or indirect cytotoxicity due to the production of reactive oxygen species upon irradiation with ionizing radiation (X-rays or γ-rays). The efficiency of radiotherapy may be improved in the presence of various inorganic NPs (gold, gadolinium, hafnium or iron oxide) in different ways [80]. Energy deposition may be improved, as high Z materials such as gold or gadolinium have a high absorption cross section for the ionizing radiation [81]; however, to obtain a significant increase compared to the surrounding water requires very high NP concentrations. Another strategy relies on using lanthanide-doped semiconductors, such as Gd/Eu-doped ZnO, to benefit from X-ray absorption from lanthanide and from the photocatalytic properties of ZnO [82]. A third mechanism of radio-sensitization is based on Fenton’s reaction. NPs with Fe^2+^ or Cu^+^ cations at their surface can be oxidized and convert the H_2_O_2_ produced by the ionizing radiation into more toxic HO∙ hydroxyl radicals [83,84]. Finally, NPs may also be used to sensitize tumor cells before irradiation by reducing the level of anti-oxidants [85], increasing the intra-tumoral concentration of O_2_ [86], or by triggering other, more complex, biological effects [87].

## 3. Designing Surface Ligands

**Figure 4:**
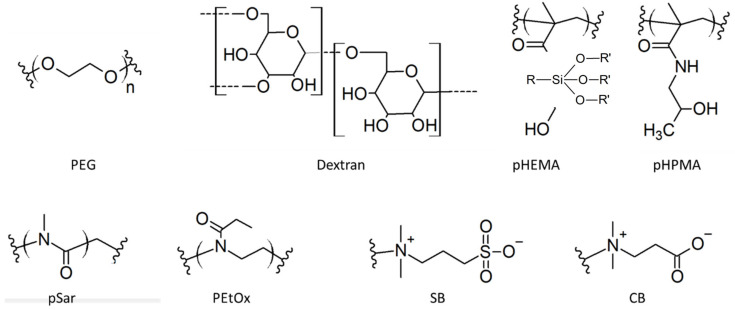
Structures of the solubilizing functions commonly used in the surface chemistry of inorganic NPs.

In this section, we present the different classes of surface chemistry that have been developed to coat inorganic NPs in the context of bio-applications and, more specifically, cancer therapy. The surface chemistry of inorganic NPs can be described following a minimal general principle. Surface ligands need (i) to interact with the NP inorganic surface, and (ii) to present hydrophilic groups to ensure water solubility. Surface ligand molecules can be present directly during the aqueous synthesis of NPs, but most often the surface ligands required during the synthesis to control growth, size and shape are not adapted to direct in vivo applications. Conversely, surface ligands that are appropriate for in vivo applications are rarely adapted for the direct aqueous synthesis of NPs. NPs are thus more generally synthesized with a set of initial ligands. When these initial ligands are hydrophobic, the NPs can be transferred to aqueous media by using encapsulation with amphiphilic molecules or polymers. The initial ligands can also be directly reacted with the final desired hydrophilic surface chemistry. The simplest strategy remains to exchange entirely the initial ligands used during NP synthesis with the final ligands. We divide the following discussion into two parts, first describing the different anchoring groups used to bind organic molecules to the surface of inorganic NPs (summarized in Table 1), and then describing the different hydrophilic moieties used to solubilize them (Figure 4).

### 3.1. Anchoring Functions

**Magnetic iron oxide NPs.** Anchoring groups binds to inorganic NP through interactions with the inorganic ions at the surface. For magnetic iron oxide NPs, these ions are Fe^2+^ or Fe^3+^. Polysaccharides, such as dextran, chitosan and starch, have been extensively used to stabilize IO-MNPs, but they only bind weakly to their surface through their hydroxyl or amine functions and can, therefore, easily detach (cf. infra). Whenever prolonged stability is sought, stronger anchoring functions are desired. Carboxylic acids, catechols, silanes and phosphonates are among the chemical functions that most effectively bind to IO-MNPs. The nature of the anchoring group not only influences ligand affinity and colloidal properties but may also influence the oxidation state of surface iron atoms and the magnetization of the functionalized particles. Carboxylic acids efficiently bind to the NP surface, especially in multidentate ligands like citric acid. They are even able to efficiently remove and replace ligands originally present on the NP surface, and can be used to transfer IO-MNPs synthesized in organic solvents to an aqueous phase [88,89,90]. However carboxylic acids are reported to negatively affect the relaxivity and saturation magnetization of IO-MNPs, thereby making them less interesting for applications [91]. Dopamine and other catechol derivatives also efficiently bind to the magnetic NP surface [88]. Particular attention should be paid to the nature of substituent groups on the catechol ring, since it directly affects the binding affinity and surface grafting density, but also the pH stability of the anchoring groups. Dopamine-derived ligands have been shown to suffer from re-protonation and desorption at lower pH conditions. In addition, they are sensitive to iron(III)-mediated oxidation, leading to particle degradation and desorption. The presence of substituents on the catechol ring can significantly improve ligand performance. In particular, 5-nitrodopamine and 5-nitroDOPA derivatives have much more stable anchoring functions than dopamine ligands. They preferentially bind Fe^2+^ surface atoms, making them suited for Fe_3_O_4_ MNPs, but not for Fe_2_O_3_ [92,93,94]. Catechol ligands advantageously preserve the spin orientation of surface atoms and retain high saturation magnetization and relaxivities [91,94]. Silane groups are also used to bind to magnetic NPs. They preserve the properties of the magnetic NPs and are robust to pH changes [95,96,97]. Phosphonates are also strong anchoring groups for IO-MNPs in biological conditions [98]. Their stability can be further enhanced in bi-phosphonate ligands [99,100]. They maintain a high relaxivity and saturation magnetization [101,102,103,104].

**Gold NPs.** To immobilize ligand molecules at the surface of gold NPs, taking advantage of the covalent Au–S bond is the most widely used technique because of the high affinity between gold and sulfur atoms. Anchoring groups that contain sulfur atoms include monodentate or multidentate thiols, which can be easily coupled at the end of polymer chains. Several factors can influence the conjugation stability between gold and thiol, including time, temperature, pH, and the concentration of salts. To avoid the dissociation of thiol terminated molecules from the gold NP surface, functionalized gold NPs should be stored in a relatively low salt, neutral or slightly acidic buffer, at low temperature [105]. Ligands containing several thiol groups, such as dihydrolipoic acid derivatives, provide more long-term stable functionalization.

Another functionalization agent that has recently been applied to bind polymers on the surface of gold NPs is N-heterocyclic carbenes (NHCs). Due to their strong σ-donation, NHCs can strongly bind to gold atoms by forming highly robust complexes [106]. Sherman et al. [107] proved that NHC ligands remain intact on gold NPs for a period of 21 days without dissociation under different conditions, such as PBS buffer, tris-glycine potassium buffer, tris-glycine potassium magnesium buffer, cell culture media and human serum. Nosratabad et al. [108] synthesized NHC-terminated PEG ligands (NHC-PEG). NHC-PEG functionalized gold NPs are stable for a period up to one year in different conditions (pH range 3–8, NaCl 1 M). However, in the presence of competing small molecules such as dithiothreitol (DTT), the colloidal stability of such NHC-PEG functionalized gold NPs decreases, which can be explained by dislocation and reorganization of NHC-bound gold atoms. Therefore, by combining two different anchoring groups, the thiol group and the NHC group, one could possibly prolong the colloidal stability of gold NPs in complex biological environment [109]. Aryldiazonium salts (N = N^+^-Aryl) have also been utilized as gold surface anchoring groups in recent decades. Briefly, the diazonium group (N = N^+^) can attach with gold atoms at the NP surface, then the diazonium group can be reduced into nitrogen molecules and be eliminated. Finally, stable Au-C covalent bonds are produced [110]. It has been proved that the aryldiazonium salts are capable of replacing the cetylammonium bromide (CTAB) surfactant around gold nanorods, maintaining the colloidal stability of NPs, at the surface of gold nanorods by a simple ligand exchange process in water solution [111]. Knowing that CTAB is indispensable during gold nanorod synthesis, while it induces severe cytotoxicity, clearance of CTAB surfactant is always a key step for bio application of gold nanorods. Compared with thiol groups, aryldiazonium salts possess advantages such as higher stability under harsh conditions and better versatility of post functionalization [112].

**Quantum dots.** Semiconductor quantum dots used in biomedical applications are usually terminated by a shell of ZnS to passivate electronic surface traps and improve their biocompatibility. Most ligands bind the surface Zn^2+^ ions. For example, carboxylic acids, such as oleic acid, or phosphonic acids, such as octadecylphosphonic acid, provide ligands which bind to Zn^2+^ in their deprotonated forms and are commonly used as surface ligands during QD synthesis in nonpolar media. These groups could be used to stabilize QDs in water at high concentrations [113], but bind zinc ions too weakly to provide stability in biomedical applications. Thiolates are binding zinc surface atoms with a higher affinity. Monothiol ligands such as mercaptopropionic acid or glutathione can be used as labile ligands but still desorb rapidly. The multiplication of thiol anchoring groups in the same molecule is an efficient strategy to increase ligand stability and limit desorption. Dithiol molecules such as dihydrolipoic acid derivatives provide more stable anchoring [114,115], which may be sufficient for in vitro applications, but eventually desorb from the surface, especially under dilute conditions [116]. Ligands with three [117] or four [118] thiol groups, or peptides with multiple cysteines [119] are even more stable. These thiol-based ligands are relatively simple to use but are not efficient under acidic (typ. pH < 4–5) conditions where the thiolates re-protonate into thiols. They are also sensitive to (photo)oxidation and may form disulfide bonds which then lose their affinity for the QD surface [120]. L-type ligands may also be used, such as phosphines [121], in nonpolar solvents but not in aqueous media. Imidazole-containing polymers [122,123], and poly(histidine) tagged peptides [124,125], proteins [126] and oligonucleotides [127] can also efficiently bind QDs. In contrast to thiol-based ligands, imidazole ligands are insensitive to oxidation; however, they remain sensitive to protonation under acidic conditions (typ. pH < 4–5).

In addition to selecting the chemical function with the best affinity for the inorganic surface, a recurring strategy to enhance stability is the multiplication of anchoring groups on the same molecule, to benefit from a multidentate effect. Adding several anchoring groups to the ligand increases its affinity for the NP surface [128]. These multiple groups can be present within the same molecule, e.g., as neighboring thiol functions in dihydrolipoic acids, or in poly(cysteine) or poly(histidine) peptides. They may also be incorporated randomly in statistical polymers, where they are interspaced with other monomers used to promote water solubility or grouped together in block copolymers. Statistical polymers are usually easier to prepare, but the presence of anchoring functions in the outer polymeric layer may induce undesired nonspecific interactions with biomolecules. In addition, the stability of polymers seem to be enhanced when the anchoring groups are grouped as a block, compared to a random statistical distribution [129].

Finally, another strategy that has been used to functionalize the surface of many NPs involves coating them with a thin layer of silica. Siloxane molecules with one of the anchoring functions mentioned above for the specific NP types can be deposited on the NP surface [130,131] or, in the case of oxide NPs but also with most QDs, Si-O- groups, can directly bind to the surface atoms [132,133]. Another strategy is to first coat the NPs using an appropriate polymeric layer with an affinity for both the NP surface and silica, such as polyvinylpyrrolidone [134]. Then silica can be deposited onto these NPs using hydrolysis and condensation of silica precursors such as tetraethoxysilane. Even though the anchoring stability of one silane molecule is weak, the silica forms a network of cross-linked covalent -Si-O-Si- bonds, several nm in thickness, which prevents desorption from the NP surface. The silica layer is always porous to water and protons, and larger nanometric pores can be created to encapsulate small molecular drugs [135]. Then the surface of the silica layer can be functionalized with appropriate silane to coat the NP with the different solubilizing polymers described above (PEG [136], zwitterions [137,138]) One of the limits of this approach is the continuous hydrolysis of Si-O bonds, which leads to stripping of surface functionalization molecules and to silica dissolution, especially under diluted conditions and in the biological environment [138]. It also increases the overall NP size, which can impact their biodistribution. The silica shell coating may degrade the luminescence of QDs. When deposited onto AuNPs, it improves the stability of the AuNP shape, as it prevents gold nanorods or nanostars from turning into spheres under high power illumination during photothermal therapy [139]. However, it reduces the temperature increase at the NP surface, which could impact the photothermal efficiency.

### 3.2. Hydrophilic Functions

The solubilizing part of the surface ligand controls the colloidal stability of the NP in the aqueous environment. Indeed, when NPs are resuspended in water, they are attracted to each other due to Van der Waals forces arising from the difference in dielectric constant between the NP material and the surrounding solvent. The first role of surface ligands should be to counteract this attraction to prevent agglomeration. For this, charged groups, such as carboxylates in citrate-capped iron oxide NPs may provide electrostatic repulsion to stabilize the NPs. However, the high salinity of physiological media provides counterions to screen this repulsion, making it ineffective. pH changes may also modify the charge of the ligands, depending on the pKa of the ionizable groups. Finally, charged NPs are more susceptible to nonspecific interactions with proteins or other biomolecules, with a generally detrimental effect on their stability in complex biological media such as blood. NPs should, therefore, preferentially remain neutral overall in order to avoid these nonspecific interactions. The stability of most biologically relevant NPs relies on the steric hindrance provided by neutral polymeric ligands. Hydrophilic polymers anchored on NPs form a hydrated layer around them, with water molecules more or less strongly bound to them, by means of either hydrogen bonds (PEG, pHPMA, Peptoids, Dextran) or ionic structuring (zwitterions). The polymers provide effective steric repulsion between NPs, arising from the fact that the entropy of the surface polymers is higher when polymers are extended and surrounded by small solvent molecules instead of becoming compressed in between two agglomerated particles. This repulsion is more effective when the surface density of the polymeric ligands and the polymer molecular weight are higher [140]. The polymeric architecture (e.g., linear vs. branched) may also influence this entropic stabilization. Finally, depending on the nature of the polymer and its interaction with water molecules, this stabilization may be affected by salt and temperature, as in the case of polyethylene glycol [141]. In the following section we discuss the different hydrophilic functions most commonly used to solubilize inorganic NPs, with their structures represented in Figure 4.

Polyethylene glycol (PEG), a group of polymers composed of ethylene glycol monomers (-O-CH_2_-CH_2_-O-) with various molecular weights, is currently the most employed stabilizing ligand for inorganic NPs in biological media. It is believed that the high water solubility of PEG arises from the partial negative charge located on the oxygen atoms, which attracts the partially positively charged hydrogen atoms from water [142]. Thanks to its hydrophilic nature, PEGs grafted on the surface of inorganic NPs can form a hydrated cloud with a large excluded volume that prevents NP aggregation and protein adsorption [143]. Its hydrophilicity, however, decreases as temperature and salt concentrations increase [144]. PEG molecular weight is considered an important factor that determines the anti-fouling effectiveness of the PEGylation layer. A value between 1500 and 5000 Da is suitable for the required stealth property [145]. Apart from molecular weight, the surface density of the PEG layer is another critical factor for resistance to protein adsorption. When the surface PEG density is low, one PEG molecule doesn’t overlap with its adjacent PEG chains and prefers to take a “mushroom” conformation. As the surface PEG density increases, the neighboring PEG chains overlap and are forced to stretch away from the surface of the NP, leading to a “brush” conformation. For example, PEGylated gold NPs have been applied in cancer theranostics [32,146,147]. It is believed that surface PEG densities in the mushroom-to-brush transition usually ensure the necessary resistance to protein adsorption and reduce the uptake of NPs by macrophages [148,149]. By utilizing heterobifunctional PEGs (HS-PEG-NH_2_, HS-PEG-COOH), different kinds of molecules, including antibodies, proteins and fluorescent probes, can be immobilized on the surface of gold NPs [32,150,151]. As for iron oxide NPs, recently it was found that the property of the PEG layer has an even more important effect on the half-life time than the size of NPs by significantly determining the clearance ratio of NPs [152]. Although PEGylated NPs possess anti-fouling properties, the hydrophobic blocks in PEGs, such as the backbone (-CH_2_CH_2_-) and methoxy terminal (-OCH_3_), can still induce nonspecific interactions and provoke an immune response and a subsequent production of anti-PEG antibodies [153]. As a consequence, repetitive injection of PEGylated NPs can eventually cause accelerated blood clearance (ABC) due to fast recognition by these antibodies, eventually leading to a poor NP biodistribution [154]. Finally, it should be noted that PEG is known to slowly degrade in oxidative conditions [155].

Dextran is a polysaccharide that is commonly used to coat several clinically-approved iron oxide NPs, such as Feridex/Endorem, Sinerem/Combidex and Resovist/Cliavist [43,44]. It is a ramified polymer composed of glucose units which can be further modified with carboxylic acid, amine or sulfate groups. These polymers interact via multiple weak interactions with the surface of iron oxide NPs. To avoid desorption of the polymer from the surface after injection in a biological fluid, the polymer can be chemically cross-linked to form a stable covalently bound coating around the nanocrystal [156]. QDs have also been coated with dextran to target macrophages in vivo [157]. Dextran is a ligand for lectin receptor-mediated endocytosis through DC-SIGN and L-SIGN receptors, which are expressed on phagocytotic cells such as macrophages and dendritic cells [158,159].

Poly (N-(2-hydroxypropyl)methacrylamide), pHPMA, is one of the first polymers tested for passively tumor-targeted drug delivery in clinical trial [160] and is widely used in nanomedicine developments for drug delivery, imaging and combination therapy [161]. It usually improves the circulation times of drugs, as well as their biodistribution. pHPMA and, to a larger extent, poly(2-hydroxyethyl methacrylate), pHEMA, a polymer with a similar architecture, are both used as hydrogels in biosensor applications. They have also been used to coat inorganic nanocrystals for biological applications. They have often been used as a hydrophilic coating in combination with other functional polymers. For example, iron oxide NPs have been coated with pHEMA grafted with biodegradable poly(ɛ-caprolactone) (pCL). The drug chlorambucil was loaded in the iron oxide polymer coating by interaction with pCL, and could be slowly released in solution [162]. In another study, a copolymer was prepared from hydrophilic pHEMA and hydrophobic perfluorophenyl azide (PFPA)-derivatized HEMA to form an amphiphilic copolymer able to self-assemble as stable micelles encapsulating iron oxide NPs and QDs [163]. Another example is the use of HEMA in a copolymer with dopamine methacrylamide to coat IONPs, where HEMA provides water solubility while dopamine enables anchoring to the IONPs as well as pH sensitivity. The obtained NPs were envisioned as pH-sensitive drug carriers and magnetic hyperthermia probes [164]. However, reports on the uptake of pHEMA-coated IO-NPs by mammalian cells suggests some degree of nonspecific interactions with cell membrane components [165] and, to date, the in vivo pharmacokinetics of pHEMA or pHPMA-coated inorganic NPs have not been studied.

Peptoids, a class of polymers analogous to peptides, represent a promising alternative to PEG. In these polymers, monomers are also bound via amide groups, but side chains are bound to the nitrogen instead of the carbon atom on the backbone. They have been shown to minimize nonspecific protein adsorption on NPs and, in contrast to PEG, to be non-immunogenic [166]. For example, NPs coated with poly(2-ethyl-2-oxazoline), PEtOx, show the same minimal level of nonspecific protein adsorption and cellular uptake as with PEG, making them a promising candidate for clinical applications [167]. Peptoid poly(oxazoline) and poly(sarcosine) ligands have been developed to coat iron oxide NPs, gold NPs and QDs [168,169,170,171,172,173,174,175]. For example, IONPs coated with PEtOx and loaded with paclitaxel have been proposed for magnetically guided cancer therapy [170]. In another example, gold nanorods coated with PEtOx and loaded with doxorubicin were evaluated in vitro for combined chemo and photothermal cancer therapy [171]. Polysarcosine has been also tested to coat gold nanorods. They showed a slightly longer blood circulation time and improved tumor uptake compared to PEG-coated AuNRs, and led to a similar PTT efficacy in nude mice bearing xenografts tumors [174].

Zwitterions contain two groups of opposite charges separated by a short linker, similar in structure to the polar headgroups of many phospholipids present in cellular plasma membranes. Typical examples of zwitterions with biomedical applications are sulfobetaine, carboxybetaine, phosphorylcholine and amine oxides. Their high charge density strongly attracts water molecules and makes them highly hydrophilic. In contrast to polyelectrolytes, they become more soluble as the ionic force increases in the solution, making them highly stable in biological media [176]. At the same time, they carry a zero net charge, which prevents long range electrostatic interactions. In general, longer alkyl linkers between the two opposite charges decrease the overall hydrophilicity of the zwitterion and increase hydrophobic interactions with biomolecules. These hydrophobic interactions are, however, virtually nonexistent with 1- or 2-carbon linkers [177]. Some more or less specific interactions might remain with specific zwitterion/protein pairs, such as phosphorylcholine and C-reactive proteins [178,179], but overall these properties make zwitterions efficient anti-fouling coatings, since they do not bind to most proteins [180,181]. They are thus commonly used as substrate coatings to prevent protein or cellular adsorption [182]. They are also conjugated to protein drugs to limit recognition by the immune system and prolong biological availability after intravenous injection. In contrast to PEG, zwitterionic polymers do not elicit an immune response, so do not exhibit accelerated blood clearance [153]. These attractive properties have recently motivated their application to NPs [183]. Gold NPs were among the first NPs coated with zwitterions [184,185,186], and their surface chemistry has been extended to other NPs such as silica [137,138], iron oxide [187,188] and QDs [123,189,190]. Usually zwitterionic polymers are used to coat NPs, but single molecular layers can also be used to obtain very compact NPs [188,189]. Compared to NPs coated with PEG or dextran coating, their small sizes may be advantageous to obtain renal excretion of NPs into the bladder or facilitate their diffusion in cells and tissues. NPs coated with zwitterions have been shown to be extremely stable under a broad range of pH, salinity and protein concentrations.

After functionalization with surface ligands, several methods may be used to analyze the composition of the surface chemistry, such as Fourier Transform Infrared spectroscopy and nuclear magnetic resonance, in order to identify and quantify the molecules bound to the nanocrystal surface [191,192]. Elemental analysis using X-ray photoelectron spectroscopy, energy dispersive X-ray spectroscopy, mass spectroscopy or atomic emission spectroscopy may also provide quantitative information about the ratio of the various elements present in the NP itself and heteroatoms (N, P, S) in the surface chemistry ligands. Thermogravimetric analysis (TGA) gives access to the mass ratio between surface organic molecules and the inorganic NP. This is helpful to estimate the surface density of polymer layers. The stability of the ligands on the NP surface is a critical parameter, which can be tested by characterizing NP stability under dilute conditions, to favor ligand desorption, or by directly monitoring the desorption of a ligand by NMR or optical spectroscopy if the ligand is labeled with a fluorophore. The colloidal properties of the obtained NPs are also typically characterized using dynamic light scattering and zetametry in biologically relevant pH and salinity conditions before studying their behavior in the presence of biomolecules.

## 4. Interactions with Surrounding Biomolecules: The Protein Corona

### 4.1. Formation of the Protein Corona

**Figure 5:**
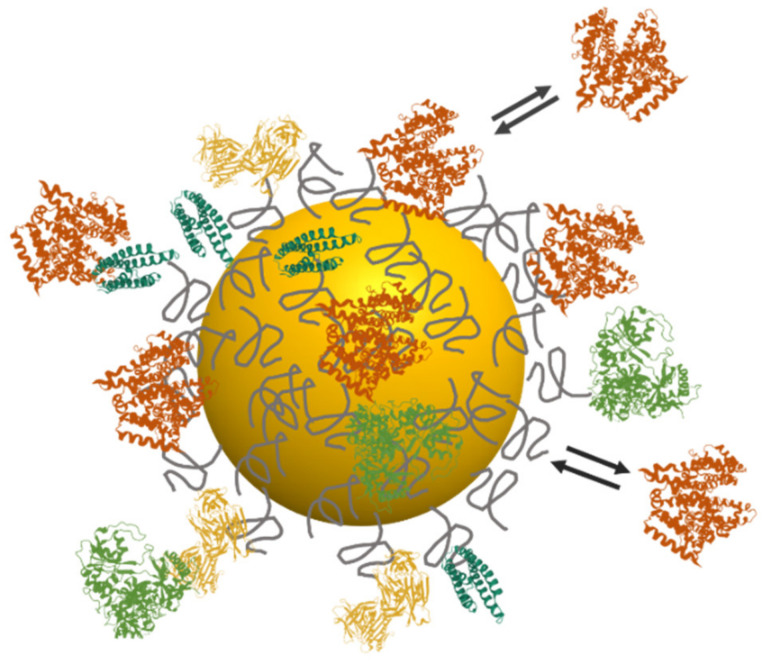
Schematic showing an NP surrounded by surface organic ligands and coated with various strongly bound proteins (hard corona) and weakly bound proteins in equilibrium with proteins in solution (soft corona).

After subcutaneous, intramuscular or intravenous injection, the NPs are dispersed in biological fluids (blood, lymph) with a complex composition of ionic species, proteins, lipids, and small molecules. The NP stability in the presence of ions can be easily characterized in vitro and is a prerequisite before in vivo applications. The interactions with proteins are, however, much more difficult to predict, due to the vast variety of protein composition and conformation. Proteins may interact directly with the inorganic surface of the NP if the ligand coating is loose or not dense enough to prevent protein access to the inorganic surface. They also interact with the surface chemistry coating through hydrophobic, electrostatic, or hydrogen bonding interactions. The strength of NP-protein interaction is highly dependent on their respective chemical and physical properties. Weakly bound proteins are in a dynamic equilibrium between bound and unbound states and form the so-called “soft corona”. On the other hand, strongly bound proteins that resist washing and purification procedures form the “hard corona” (Figure 5). This protein corona plays a major role in the biological fate of the NPs, as it determines the interaction of NP with other biomolecules and cells [193,194]. The composition of the protein corona is highly dynamic and evolves over time [195]. This phenomenon is known as the Vroman effect and has been explained by the proteins being more concentrated and/or diffusing faster first access the surface of the NP to form the initial protein corona. They are then replaced by proteins with higher affinities, which are more stably anchored on the NP surface. When a protein is anchored on the NP surface, it is susceptible to undergoing conformational changes to optimize its interaction with the NP. This denaturation can, in turn, expose hydrophobic segments which were initially within the protein structure and now become accessible for further interactions. These conformational changes may lead to novel, potentially stronger protein-protein interactions, leading to cooperative NP-protein and protein-protein interactions, to formation of protein multilayers, or to NP aggregation. The structure of the NP-protein complexes also depends on the relative and absolute concentrations of NP and proteins. For example, strong interactions between an NP and a protein may lead to NPs surrounded by a protein corona if the protein is in large excess. However, if these proteins are in a low concentration, they may bind to several NPs and lead to the formation of large NP clusters coexisting with protein-free NPs. The protein corona, therefore, affects the NP physico-chemical and colloidal properties, by changing their colloidal stability, hydrodynamic size, and zeta potential.

### 4.2. Analytical Methods to Characterize the Protein Corona

There are several methods to study the protein corona. They can be divided into two groups, with either a direct detection of proteins or molecules, or an indirect detection, where a change in NP properties is detected. Direct methods are used to identify proteins or biomolecules, quantify them or have access to structural information. Gel electrophoresis can be used to identify biomolecules. In gel electrophoresis the denatured proteins are separated based on their size and charge. In the case of a complex mix of proteins it is not possible to absolutely identify proteins based only on their electrophoretic mobility. Negative staining with transmission electron microscopy can also be used to check whether proteins are adsorbed on the surface, but it is not quantitative. Other methods are used to quantify proteins without identifying them, such as widely used protein assays that quantify the number of proteins in solution (Bradford assay, bicinchoninic acid, and fluorescamine assay). Inductively-coupled plasma mass spectrometry (ICP-MS) with inorganic NPs can estimate accurately the ratio of inorganic ion from the NPs compared to the sulfur of proteins, providing a precise ratio of proteins per NP. Beyond quantifying the amount of proteins around each NP, reliable identification of the composition of the protein corona is often achieved using advanced mass spectrometry techniques such as tandem mass spectrometry (MS/MS), matrix-assisted laser desorption/ionization time-of-flight mass spectrometry (MALDI-TOF MS) or liquid chromatography coupled to mass spectrometry (LC-MS) [196,197,198,199]. In addition to detecting which proteins bind to the NP, circular dichroism or Fourier transform infrared spectroscopy (FTIR) can be used to monitor structural changes in their conformation. All these methods can give relevant and useful information about the protein corona. Nonetheless, they all require a washing step to separate unbound proteins from the NPs. The washing method can influence the results; when centrifugation and magnetic separation method were compared, only 50% of the protein detected in the protein corona were common to both methods [190]. Washing also changes the dynamic equilibrium of bound to unbound proteins, which changes the composition of the loosely attached proteins layer, or soft corona. Extensive washing can remove all the weakly bound proteins. The soft corona does, however, play an important role in vivo, and reliable direct methods still need to be developed to characterize its composition in situ [200,201]. For example, SERS (surface enhanced Raman spectroscopy) uses the amplification of the Raman signal of a molecule when it is in the immediate proximity of the surface of a metal NP [202]. Proteins in the solution that are not bound to NP are not detected, so the washing step is not required, and the equilibrium of protein binding can be conserved.

There are also many indirect methods to study the protein corona. Most of them do not require washing, which is useful to study both the hard and soft corona. These methods rely on the change of a property of the NPs such as a change in size, charge, density, or mass. Change of size is widely used, and can be either directly observed with microscopy techniques such as electron or atomic force microscopy, or via a change of Brownian motion due an increase of hydrodynamic size. This includes techniques such as NP Tracking Analysis (NTA) [196], Dynamic Light Scattering (DLS) [203], and Fluorescence Correlation Spectroscopy (FCS) [181]. DLS and FCS can detect smaller particles than NTA. DLS is, however, not effective in the presence of large concentrations of proteins similar in size to NPs, since it becomes difficult to discriminate the signal from the large excess of free proteins from the signal of the NP. Depolarized DLS can overcome this issue with anisotropic NPs, since the signal from the proteins can be subtracted from the NP signal because they do not exhibit depolarized scattering [204]. ^19^F diffusion-ordered nuclear magnetic resonance (NMR) spectroscopy can also be used with ^19^F labelled NPs [205]. Here the measure of diffusion does not rely on visible light, so it can be performed even in turbid environments and in the presence of cells. Gel electrophoresis or size-exclusion chromatography (SEC) can reveal a change of size due to the protein corona. Binding of macromolecules change the sedimentation coefficient, which can be measured by Differential Centrifugal Sedimentation (DCS) [206]. Formation of a protein corona can also result in a change of charge at the surface of the NPs that can be followed with capillary electrophoresis (CE) where particles are displaced in an electric field based on their electrophoretic mobility, which depends on both the size and charge of the NP [207,208]. Changes in mass due to protein adsorption can be detected with extreme precision using a Quartz Crystal Microbalance (QCM). NPs are fixed on an oscillating quartz plate, and the frequency of oscillation changes when a protein is adsorbed on an NP, thus measuring its mass [209,210]. Comprehensive reviews on the characterization of the NP protein corona are available for the reader (see e.g., [211,212]).

All these methods are useful for studying the protein corona. Because they give complementary information in term of number, protein type, conformation it is interesting to use several methods to gain a better picture of the nature of the protein corona. Conditions in which the protein corona is studied also play an important role. For example, biological solutions are not equally relevant: circulating whole blood would give more relevant results than blood serum which is depleted of some proteins and coagulation factors. The same NPs can have a very different protein coronas in in vitro and in vivo experiments [213]. Particle and protein concentrations each play a role in the composition of the corona. It should also be kept in mind that the corona is not only formed of proteins but also, usually, of other biomolecules such as lipids [214]. Finally, the exact blood plasma composition varies from patient to patient and is believed to depend on the health status of the patient. Thus, the composition of the protein corona may reflect different pathological statuses, giving rise to the concept of a personalized protein corona [215].

Clearly, the nature of the surface chemistry strongly influences both the nature and the strength of NP-protein interactions [216]. The composition of the protein corona, the amount of strongly or weakly bound proteins and the overall architecture of the corona depend on a large number of parameters conferring the NP’s physico-chemical properties. General principles may be recovered from the vast number of studies devoted to protein corona formation around NPs. A more hydrophobic NP surface favors strong van der Waals interactions, leading to strongly bound proteins displaying high conformational changes. A positively charged NP surface favors electrostatic interactions with proteins, which are usually negatively charged overall at physiological pH. However, negatively charged NPs also strongly interact with proteins, which can exhibit positively charged local surface patches. Globally a neutral NP surface chemistry is preferred to limit these interactions. Hydrogen bonding also participates in NP-protein interactions, whereas highly hydrophilic NP surfaces with strong hydration layers, such as zwitterionic surfaces, may limit these types of interactions since it becomes more difficult for biomolecules to replace water molecules surrounding the surface molecules. Polymer density also plays an important role, since a polymeric coating that is too loose may enable direct interactions between proteins and the NP surface. In particular, in the case of polyethylene glycol, but probably also for other polymers, the polymer density dictates its conformation as a “mushroom” or “brush” layer, which interacts differently with surrounding biomolecules [217]. The vast variety of proteins present in biological fluids, and the complexity and dynamic nature of NP-protein interactions makes it very difficult to predict exactly the importance and the structure of the protein corona, leaving many questions open such as: is it possible and desirable to completely suppress the protein corona? and is it possible to characterize it in a way which is relevant to fully understand further interactions with cells and organisms? These questions are critical for clinical applications of NPs since, as the next section shows, the protein corona plays a central role in the biodistribution, elimination, toxicity, tumor uptake and therapeutic efficiency of NPs.

## 5. In Vivo Biodistribution and Clinical Outcomes

### 5.1. Circulation in Blood and Elimination

As discussed above, once inorganic NPs are administrated into the bloodstream they can interact rapidly and, potentially massively, with plasma proteins, leading to the formation of a protein corona. The biodistribution of the NPs is then not determined so much by the composition of the inorganic core itself (e.g., gold, iron oxide) but more by the physical size and chemical nature of the NP-surface ligand-protein corona ensemble, which can possibly be aggregated as a larger object [218]. As discussed above, the interactions between NPs and molecules from biological fluids are complex, very sensitive to experimental conditions and difficult to fully characterize. In addition, very few studies have compared side by side the pharmacokinetics and biodistribution of NPs by modifying systematically physico-chemical parameters one by one. As a result, when comparing different studies, it is difficult to draw strong correlations between the NP surface chemistry and their pharmacokinetics, due to differences in particle size, preparation and purification methods, animal model used, surface ligand nature, and density, among others. However, general principles can be drawn, which we discuss in the following section.

As a general rule, NPs with total hydrodynamic diameters smaller than 5–6 nm may undergo renal glomerular filtration, ending up in the bladder and excreted with the urine [219,220]. Even though the diameter of the NP may be initially small enough to undergo renal excretion, increase in size due to the protein corona may prevent this filtration. As a second physical size filter, NPs or NP aggregates larger than 200 nm may become sequestrated within the smallest capillaries in the spleen and liver and quickly removed from circulation by resident macrophages [218]. Similarly, large or aggregated NPs may obstruct blood capillaries in the lungs, possibly even leading to pulmonary embolism [221]. Beyond this effect of physical size, the nature of the protein corona has a strong impact on the circulation time of NPs. The protein corona may contain certain types of protein (immunoglobulins, complement proteins, and blood clotting factors), named opsonin proteins [222]. By recognizing these opsonin proteins bound to the surface of NPs, cells of the mononuclear phagocyte system (MPS), such as macrophages, can capture and take up NPs through phagocytosis, which results in the clearance of NPs from the bloodstream [223]. The clearance of NPs by macrophages is quite efficient; up to 95% NP can be finally eliminated regardless of NP nature [224]. Moreover, the recognition of foreign materials can locally activate tissue resident macrophages, which are normally quiescent, provoking the rearrangement of their surface receptors, making them even more sensitive towards NPs and leading to abnormally accelerated internalization and clearance rates [225]. Four specific macrophage surface receptors are believed to be mainly responsible for NP recognition: (1) toll-like receptors, (2) mannose receptors, (3) scavenger receptors, and (4) Fc receptors. It has been suggested that Fc and mannose receptors may play a more important role in particle uptake process than the others [225,226]. Since, in most cases, NPs circulating in the bloodstream are coated with a protein corona, there are more chances for macrophage surface receptors to interact with the adsorbed proteins than with the bare NP surface. Therefore, opsonin proteins, which are electrostatically or conformationally appropriate for specific cell receptor recognition, can largely enhance and accelerate NP uptake and clearance. By contrast, in some cases adsorbed proteins, referred to as “dysopsonins,” can mask materials from surface recognition, endowing “bioinvisibility” or stealthiness to nanomaterials [225,226,227]. For example, by tuning the hydrophobicity of cationic gold NPs, Saha et al. showed that different proteins were present in the protein corona. The presence or absence of some proteins correlated positively with the uptake by macrophages (=opsonins) while some others correlated negatively (=dysopsonins) [228]. Several apolipoproteins have been observed to coat NPs and mask them from cellular uptake. For example, when incubated in serum, PEGylated NPs become coated with different proteins, such as apolipoprotein J or clusterin. The presence of these proteins at the surface of the NPs decreases substantially their uptake by macrophages [229]. Other apolipoproteins have also been shown to reduce cellular uptake of NPs, such as ApoA4, ApoC3, while others increase it [230]. Albumin, the most abundant protein present in blood serum, can also decrease macrophage uptake of some NPs, and it has been proposed that interactions between PEG and albumin may explain the prolonged circulation time of PEGylated NPs [231,232]. However, this does not seem to be valid in every situation, as incubation of other NPs with albumin may increase their cellular uptake [229]. This seems to indicate that the effect of a specific composition of the protein corona on macrophage recognition may depend on the nature of NPs and the exact protein conformation in the corona. Overall, controlling the composition of the protein corona to exclude opsonins and favor dysopsonins seems an interesting strategy, even though it might be difficult to characterize precisely which conformational parameter is responsible for the effect, and to ensure a long-term protective effect, given the dynamic nature of the protein corona.

In order to reduce NP recognition and elimination by macrophages and prolong NP blood circulation time, several other techniques have been developed (see Table 2). The most classical and the most applied strategy is surface functionalization of NPs by hydrophilic polymers to minimize or even remove the protein adsorption at the NP surface, as discussed above. One possible method for prolongation of NP circulation is to saturate the clearance capacity of the MPS. For example, by systematically injecting a trillion gold NPs in mice, a dose above which the Kupffer cells in the liver are overwhelmed, NP clearance was significantly decreased and up to 12% of injected NPs finally reached tumor cells [233]. In addition, instead of saturating the MPS by overdosed NPs, one can force the MPS to intensify the clearance of the organism’s own intact blood cells, such as erythrocytes, by injecting allogeneic anti-erythrocyte antibodies, leading to an NP clearance blockade [234]. Another way to prolong NP circulation time is the encapsulation of NPs by red blood cells, which act as biocompatible carriers, a concept called cell hitchhiking. Magnetic NPs [235] or silica NPs [236] internalized by or attached to red blood cells have proved to be able to escape from the recognition of MPS in the liver, and these NPs can be retained afterwards on the cell surface by electrostatic interactions. Instead of using whole cells, NPs may be coated by lipid and protein patches coming from the membranes of lysed red blood cells [237].

Macrophages possess signaling pathways to inhibit phagocytosis of cells expressing the membrane protein CD47, which works as a “marker of self”: it signals its identity to the macrophage and prevents clearance from the bloodstream. Using this natural mechanism, NPs functionalized with a minimal CD47-based “self” peptide circulate longer than nonfunctionalized peptides [238].

When NPs are not immediately taken up by macrophages from the liver and spleen, they can circulate and eventually accumulate in other cells and organs. They may extravasate and infiltrate tissues where blood vessel endothelial cells do not form a tight barrier, in which case they can target specific cell populations or be captured by resident macrophages and transported into lymph nodes. This transport enables easy labelling of regional lymph nodes after subcutaneous injection, with efficiencies that also depend on the surface chemistry and nonspecific interactions with the environment [57]. They may also bind to specific membrane receptors (see below). Small (<150 nm) NPs may also extravasate through the liver endothelial barrier into the space of Disse, where they may be endocytosed by hepatocytes. NPs can then potentially be transported to the bile duct by transcytosis, leading to final excretion in the feces [220,239]. This excretion route is however slow (days to months) and is rarely completely efficient.

Since surface chemistry controls NP biodistribution, NP toxicity is directly correlated with surface chemistry: accumulation of NPs in the lungs, kidneys, heart, brain etc. can induce a higher toxicity than in the liver. Not only organ distribution but also the types of cells (e.g., macrophages, endothelial cells, nerve cells) which have engulfed NPs and the subcellular localization (membrane-bound, endosomes, lysosomes, and cytoplasm) have an important impact on NP toxicity, all of which are dependent on the NP surface chemistry and the protein corona [240]. It should also be noted that the coating itself may induce NP toxicity, even with iron oxide NPs that are considered to be very biocompatible [241]. NP toxicity may be caused by various mechanisms. NPs may induce oxidative stress and generate radical oxygen species by redox processes [242]. All these effects are surface-mediated; beyond biodistribution, surface chemistry has a crucial role in determining NP toxicity [243]. For example, weakly bound surface ligands quickly expose the NP surface and trigger detrimental effects, while strongly bound ligands may prevent or slow down interfacial redox processes.

Dissolution of inorganic NCs leads to the release of metallic ions, which can be more or less toxic depending on NC composition. The choice of a biocompatible NP composition and understanding of its in vivo degradation is, therefore, of prime importance [244,245]. In general, the size of the NP impacts the dissolution kinetics; as smaller NPs have a bigger surface to volume ratio, dissolution is faster. In the case of QDs, the dissolution kinetics depend strongly on the QD inorganic materials used. Some QDs (Ag_2_S or those coated with a ZnS shell, in particular) may degrade very slowly over several years [246], while others (core-only QDs in particular) may degrade very quickly over a few hours [247]. Fast dissolution releases a burst of metal ions which can lead to acute toxicity, in particular in the case of Cd^2+^, Pb^2+^ or Hg^2+^-containing QDs. More stable QDs release their contents over a longer period, diminishing the risk of acute toxicity but increasing the risk of long-term toxicity, especially in the case of repeated administration. The nature of the QD surface ligands also plays an important role in modulating the dissolution kinetics. For example, the surface modification of CdTe quantum dots by PEG has proven to be capable of improving biocompatibility and reducing the toxicity of quantum dots by slowing the release of Cd^2+^ [248]. Iron oxide nanoparticles are dissolved in the liver and spleen within months, releasing iron ions which are then incorporated in the body iron pool [249,250]. This dissolution occurs in lysosomes of splenic and hepatic macrophages where the pH is lower, with chelating agents [251]. Because dissolution occurs from the surface of the NP, the surface chemistry of the nanoparticle and its protein corona play a role in the dissolution speed [252]. Results suggest that with a low grafting density, the surface is more easily accessible, while a dense coating, such as a solid polystyrene layer, slows down dissolution [250,253]. Gold nanoparticles, because of their high inertness, are believed to be able to remain intact inside organs or tissues without biodegradation [240,254]. Only gold nanoparticles with a small size (d < 10 nm) are able to be partially excreted by the kidney and hepato-biliary system [255,256]. However, as recently reported, gold nanoparticles may be eventually degraded within 6 months inside the lysosomes of macrophages, where gold nanoparticles are oxidized by ROS generated by NADPH oxidase [257].

### 5.2. Tumor Targeting Strategies

**Figure 6:**
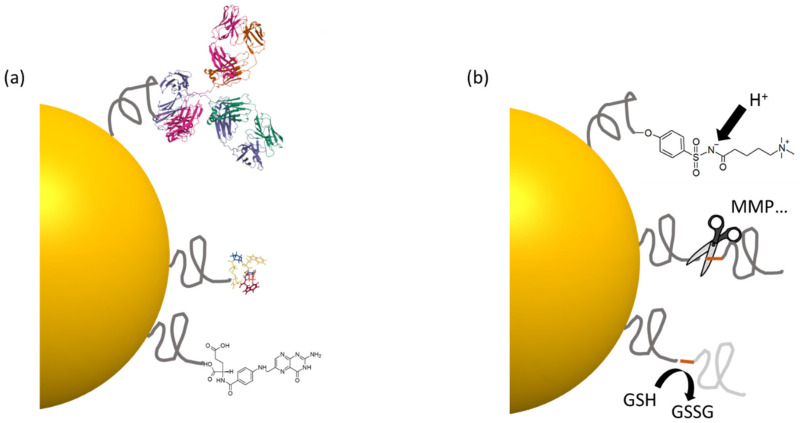
Strategies used for tumor targeting. (**a**) Active biomolecular targeting using antibodies, peptides such as RGD, or small molecules such as folic acid. (**b**) TME targeting, using acidic pH with protonable surface moieties, activity of overexpressed enzymes such as MMPs, or reduction from GSH to cleave surface ligands.

How to efficiently target NPs to specific organs or pathologies (e.g., inflammation sites, tumors) remains the topic of intense efforts. The enhanced permeation and retention (EPR) effect [258] has been largely studied and exploited for the delivery of nanomedicines, in particular in the context of cancer therapy. Tumors may develop blood vessels with endothelial walls presenting large gaps that NPs may penetrate to infiltrate the surrounding tissue. Since tumors also lack functional lymph vessels, NPs are not efficiently cleared from the tumors. Elsewhere, endothelial junctions in healthy tissues are tight, which prevents NP extravasation. As a result, in preclinical models, NPs efficiently accumulate in tumors in a passive way, with an optimal size in the range of a few tens to a hundred nanometers. However, these results fail to reproducibly transpose in human patients [259]. This is attributed to the poor blood flow within tumors and high interstitial fluid pressure, which prevents efficient delivery of NPs within the tumor volume, and to the large difference observed between tumors in human patients and tumors in small rodents preclinical models in terms of tumor relative size, permeability and architecture of tumor vasculature [260,261]. As a result, the efficiency of the EPR effect is lower and much more heterogeneous in human patients, when comparing different tumors and different regions within the same tumor. Active targeting strategies are thus highly desired to improve tumor targeting efficiency (Figure 6).

#### 5.2.1. Active Biomolecular Targeting

To perform active targeting there must be high affinity of the NP for the tumor cell that is specific to avoid the targeting of non-tumor cells. A way to achieve this is to target receptors that are overexpressed or specific to tumor cells. This targeting can be done via a whole antibody or an antibody fragment, an aptamer, a peptide or a small molecule that is attached to the NP through its ligand and binds to the tumor. Antibodies have high specificity and can also be used as therapeutic agents. For example, growth factor receptors may be overexpressed and accessible at the membrane of tumor cells, so antibodies targeting growth factor receptors may be used, such as the anti-epidermal growth factor receptor (EGFR) antibody (cetuximab) [262] or trastuzumab (anti-ERBB2, Herceptin^®^). Antibodies targeting growth factors, such as bevacizumab (anti-VEGF, Avastin^®^) can also be used [263]. Antibody fragments are engineered to be smaller than antibodies while preserving a high affinity. Antibodies are efficient due to their high specificity, but they are very large and can be costly, whereas peptides are smaller and low cost. For example, the arginylglycylaspartic acid (RGD) peptide targets αvβ3 integrins, which are particularly overexpressed in the endothelial cells of the tumor neovasculature [264]. The IL-4R-binding peptide is another example of peptide targeting a membrane receptor overexpressed in different types of cancer. Aptamers are short nucleic acid sequences that can also bind to proteins overexpressed in tumors, such as nucleolin, a nucleolar protein of growing cells or tenascin-C, a glycoprotein expressed in the extracellular matrix of various tissues during development [265]. They are specific, small, but costly. Small molecules such as folic acid, which targets folate receptors, overexpressed in many cancer cells, or anismaide that binds sigma-1 receptors overexpressed in cancer cells, have many advantages: they are small, easy to bind to NPs and very low cost. However, they may not be as specific as the other strategies because the receptors they target are also expressed at low levels in other cell types.

There are many strategies to bind antibodies, peptides, aptamers or small molecules to the NPs. Non covalent binding methods such as electrostatic or hydrophobic interactions [266] are not stable enough for in vivo applications [267], so covalent binding is often used. Many covalent bioconjugation techniques exist. Most frequently, amines from surface lysines can be reacted with carboxylic acid groups on the NP ligand that are activated as reactive intermediate esters to create stable amide bonds [268]. Alternatively, amines on the NP ligand can be reacted with heterobifunctional cross-linkers presenting a maleimide function to react with thiols from cysteines on the protein to form a thioether bound [269,270]. Azide-alkyne click chemistry, and especially copper free click chemistry using strained alkynes, is also used for covalent coupling and has been proved to be an efficient alternative [271,272]. In contrast to peptides, aptamers, or small molecules which can be prepared with controlled reactive functions, coupling of whole proteins such as antibodies, is often random due to the large number of accessible lysines. This may lead to a decrease in antibody affinity if the bioconjugation site is located close to the epitope-binding region. To avoid these issues, more complex methods may be used to orient the antibody correctly at the surface of NPs, using either protein A or G adapters [273]; however, the stability of these assemblies have not been thoroughly investigated in vivo.

Despite numerous developments and research efforts, the in vivo biodistribution of inorganic NPs remains, in general, poorly controlled. Typically less than 1 or 2% of the injected NP dose reaches and remains in the tumor [243,274]. This is attributed to the different phenomena mentioned above: nonspecific interactions between NPs and proteins, leading to the formation of a protein corona, to the decrease of binding affinity to specific biomolecular targets, and to the recognition by proteins from the immune system and capture by macrophages. While the ability to precisely target specific cell receptors remains central to NP tumor delivery, other strategies may be envisioned to improve its efficiency. For example, using the specific physico-chemical properties of the tumor microenvironment may help to overcome these obstacles.

#### 5.2.2. Tumor Microenvironment

Compared with normal cells, the microenvironment of tumor cells (TME) possesses several abnormalities such as higher acidity or temperature, different redox potential and up-regulated proteins [275]. The TME can therefore be used as a stimulator for well-designed NP surface ligands that can “smartly” recognize a specific TME. In general, these ligands are stable and inert in normal cells and tissues, while they can respond actively towards changes in the microenvironment after penetrating the tumor, leading to the accumulation of NPs in tumor cells.

NPs functionalized with pH-responsive ligands have been widely studied as promising active targeting nanocarriers, as the acidity in tumor cells is usually lower than that displayed in normal cells. In a typical TME, pH values vary between 4.5 and 5.5 because of the high cellular proliferative rate, the increased efflux of intracellular protons and low blood perfusion of tumor tissues [276]. Surface ligands such as chitosan, and ionizable zwitterions such as carboxybetaine and acylsulfonamide, possess isoelectric points, so these ligands can modify the surface charge of NPs according to the environmental acidity and eventually control the assembly and disassembly of NPs [277,278,279]. They are typically neutral in healthy tissues but become positively charged in contact with the more acidic TME, which promotes their cellular uptake. Another alternative is to use pH-responsive ligands as capping agents for drug delivery based on porous NPs. Drug molecules are loaded on the surface of porous NPs and then the NP is capped with ligands that disassemble under acidic conditions. The drug molecules cannot escape from NPs during the blood circulation until the NPs enter into tumor cells and the capping ligands are removed due to protonation in acidic pH, leading to a selective drug release towards tumor cells [280].

In addition, several kinds of enzymes are overexpressed or exhibit increased activity throughout different stage of human cancers, from initiation to outgrowth of clinically relevant metastases [276]. The matrix metalloproteinases (MMPs) is a family of zinc-dependent endopeptidases enzymes which are known to be significantly elevated and expressed in human tumor cells and are able to cleave specific peptide sequences [281]. By conjugating the peptide substrate cleavable by MMP with NPs, one can generate novel nanocarriers that are selectively activated at the tumor site for further therapy and imaging [282,283]. Apart from MMPs, other enzymes, such as cathepsin B, are also overexpressed in tumor cells, which allows various enzyme-responsive NP strategies to improve inorganic NP delivery efficiency [284,285].

The TME has a different redox potential from normal tissues. Typically, in the TME, the redox couple gluthathione/gluthatione disulfide (GSH/GSSG) plays a major role in regulation of the reducing environment of the tumor cells because the concentration of GSH in the TME is 1000 times higher than that in normal tissues [286]. Therefore, the highly reducing environment in tumor cells can elicit the degradation of redox-responsive ligands of NPs and thus selectively release loaded drugs [287]. The most widely applied strategy in redox ligand design is to use the disulfide bond (R-S-S-R), which can be degraded in the presence of GSH [288]. Inorganic materials, such as manganese dioxide (MnO_2_), are also used as active targeting nanocarriers because in the TME it can be reduced to Mn^2+^ and the decomposition of NPs allows the release of drugs [289].

## 6. Conclusions

Inorganic nanocrystals, such as plasmonic, magnetic or fluorescent NPs, offer new opportunities for cancer diagnostics and treatment due to their special physical properties. The organic molecules constituting the NP surface chemistry need to be stably anchored onto the NP surface and ensure a good interface with their biological environment: maintaining colloidal stability, limiting undesired nonspecific interactions with biomolecules and targeting cancer sites or specific cellular populations. The progress made in the design of these surface ligands now enables the efficient stabilization of NPs in biological media and prevents their aggregation due to high salinity and NP-protein interactions. Several polymers can be used as the NP surface coating to minimize the nonspecific adsorption of proteins on NPs, such as PEG, peptoids, and zwitterions. They, however, probably never completely eliminate the formation of a biomolecular corona. Different strategies are in place to hinder the subsequent recognition of NPs by the immune system, such as minimizing NP-protein interactions, or taking advantages of a specific composition of the protein corona to act as a camouflage. However, the vast number and variety of conformations of biomolecules in the biological medium, as well as the dynamic nature of these interactions, hinder the perfect understanding of the interactions between the NP surface polymer and proteins. In the context of cancer theranostics, several functionalization strategies are being employed to optimize NP biodistribution to favor tumor uptake vs. macrophage elimination. While undeniable progress has been made in these directions, better control and holistic understanding of all of these processes and their interplay are required in order to maximize imaging and therapeutic performance of NPs. The development of novel NP surface functionalization will also benefit from new delivery methods using remote physical stimuli, such as the application of ultrasound to enhance endothelial permeability, the use of magnetic gradients to localize magnetic NPs, and light-sensitive NP surface chemistry to trigger cellular uptake locally in illuminated regions [290,291].

## Figures and Tables

**Table 1:** Name, structure and material compatibility of the main anchoring function using in the surface chemistry of NPs.

Name	Structure	Applicability
Carboxylate	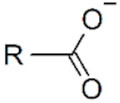	IONPs
Phosphonate	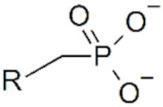	IONPs
Thiol/thiolate	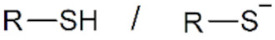	AuNPs, QDs
Imidazole	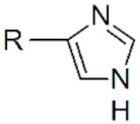	QDs
Catechol	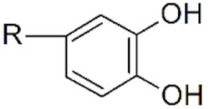	IONPs
Aryldiazonium	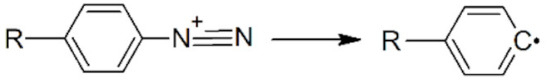	AuNPs
N-heterocyclic carbene	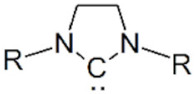	AuNPs
Silane	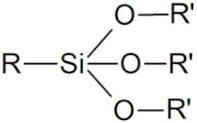	All NPs

**Table 2:** Strategies used to prolong the circulation of NPs in the bloodstream.

NPs	Applied Strategy	Model	Dose	Results	Ref.
Gold, 50 nm	Kupffer cell saturation by NPs	Mice	50 trillion NPs	Prolonged circulation time from 2 min to 8 h—Improved tumor delivery efficiency from 0.03% to 12%	[233]
Iron oxide, 100–200 nm	Kupffer cell saturation by forced clearance of erythrocytes	Mice	1.25 mg/kg of allogeneic anti-erythrocyte antibodies; Then, 25 µg NPs	Prolonged NP half-life time from ~1 min to ~15 min	[234]
Iron oxide, 100 nm	RBC-hitchhiking	Mice	200 µg NPs	Improved tumor delivery efficiency from 0.6% to 41%	[235]
Silica NP, 25–200 nm	RBC-hitchhiking	Mice	10^8^ cells	NP half-life time 3 h	[236]
Gold NPs, 2 nm	Macrophage recognition regulation by protein corona	In vitro	100 μL of AuNPs (1 μM) mixed with 400 μL of human serum	Correlated macrophage uptake induced by specific complement surface proteins	[228]
